# Behavioral Analysis and MRI Findings in a CAA Mouse Model with Chronic Cerebral Hypoperfusion

**DOI:** 10.1002/alz70856_100623

**Published:** 2025-12-25

**Authors:** Hidehiro Ishikawa, Akane Mizutani, Hideaki Wakita, Takaya Utsunomiya, Shotaro Horie, Shinobu Kitazume, Akihiro Shindo

**Affiliations:** ^1^ Mie University, Tsu, Mie, Japan; ^2^ Fukushima Medical University, Fukushima, Fukushima, Japan

## Abstract

**Background:**

Cerebral amyloid angiopathy (CAA) is linked to both dementia and stroke. Advancing our understanding of CAA pathophysiology would contribute to research in both fields. Recently, a novel mouse model expressing human amyloid precursor protein (APP770) specifically in endothelial cells (ECs) was developed. These EC‐APP770+ mice exhibit elevated serum levels of amyloid beta (Aβ) and soluble APP. The objective of this study was to investigate the impact of chronic cerebral hypoperfusion on cognitive function in EC‐APP770+ mice.

**Method:**

Six 4–6‐month‐old EC‐APP770+ mice and six wild‐type (WT) mice underwent bilateral carotid artery stenosis (BCAS) surgery. Behavioral and MRI assessments were performed 10 months post‐surgery. Cognitive function was evaluated using the Y‐maze and Novel Object Recognition Test (NORT). The experimental groups were APP‐BCAS, APP‐sham, WT‐BCAS, and WT‐sham. Behavioral data were obtained using the Ethovision XT (version 17) automated video tracking system, with Y‐maze assessing parameters such as average speed, travel distance, and spontaneous alternation behavior. In the NORT, we measured total object exploration time, exploration frequency, and the Discrimination Index (DI). MRI was conducted using a 3T‐microMRI system, including Diffusion‐Weighted Imaging, T1‐Weighted Imaging (T1WI), T2‐Weighted Imaging, T2* imaging, and Magnetic Resonance Angiography (MRA).

**Result:**

Ten months post‐surgery, APP‐BCAS mice exhibited a tendency towards decreased spontaneous alternation behavior in the Y‐maze compared to WT‐sham (*p* = 0.81), WT‐BCAS (*p* = 0.67), and APP‐sham (*p* = 0.06), suggesting a decline in working memory, though statistical significance was not reached. No significant differences in spontaneous alternation behavior were observed between WT‐BCAS and WT‐sham (*p* = 0.96) or between APP‐sham and WT‐sham (*p* = 0.39). In the NORT, the DI in APP‐BCAS mice was significantly lower compared to APP‐sham (*p* = 0.01) and WT‐sham (*p* = 0.03), but no significant difference was noted between APP‐BCAS and WT‐BCAS (*p* = 0.36). MRI analysis revealed brain atrophy in the APP group on T1WI and arterial tortuosity on MRA.

**Conclusion:**

Chronic cerebral hypoperfusion in EC‐APP770+ mice may accelerate disease progression, suggesting that the combination of CAA and hypoperfusion can exacerbate cognitive decline. Given that CAA patients often present with concomitant atherosclerotic changes, this mouse model of chronic hypoperfusion may serve as a valuable tool for studying the pathophysiology of CAA patients coexisting arteriosclerosis.

